# Vav1 downmodulates Akt in different breast cancer subtypes: a new promising chance to improve breast cancer outcome

**DOI:** 10.1002/1878-0261.12203

**Published:** 2018-05-16

**Authors:** Silvia Grassilli, Federica Brugnoli, Rossano Lattanzio, Marco Marchisio, Letizia Perracchio, Mauro Piantelli, Alberto Bavelloni, Silvano Capitani, Valeria Bertagnolo

**Affiliations:** ^1^ Section of Anatomy and Histology Department of Morphology, Surgery and Experimental Medicine University of Ferrara Italy; ^2^ Department of Medical, Oral and Biotechnological Sciences University ‘G. d'Annunzio’ Chieti Italy; ^3^ Center on Aging Sciences and Translational Medicine (CeSI‐MeT) University ‘G. d'Annunzio’ Chieti Italy; ^4^ Department of Medicine and Aging Sciences University ‘G. d'Annunzio’ Chieti Italy; ^5^ Department of Pathology ‘Regina Elena’ Cancer Institute Rome Italy; ^6^ MediaPharma s.r.l. Chieti Italy; ^7^ Laboratory of Musculoskeletal Cell Biology Rizzoli Orthopedic Institute Bologna Italy; ^8^ LTTA Centre University of Ferrara Italy

**Keywords:** Akt1, breast cancer, Vav1

## Abstract

Targeting different members of the Akt pathways is a promising therapeutic chance in solid tumors including breast cancer. The variable expression levels of Akt isoforms with opposite effects on tumor growth and metastasis, however, make it difficult to select the inhibitors to be used for specific breast tumor subtypes. Using *in vitro* and *in vivo* models, we demonstrated here that Vav1, ectopically expressed in invasive breast tumors derived cells, downmodulates Akt acting at expression and/or activation levels depending on tumor subtype. The decreased p‐Akt1 (Ser473) levels are a common effect of Vav1 upmodulation, suggesting that, in breast tumor‐derived cells and independently of their phenotype, Vav1 interferes with signaling pathways ended to specifically recruit Akt1. Only in ER‐negative cell lines, the silencing of Vav1 induced the expression but not the activation of Akt2. A retrospective analysis of early invasive breast tumors allowed to establish the prognostic significance of the p‐Akt/Vav1 relationship. In particular, low Vav1 levels negatively influence the follow‐up of patients with low p‐Akt in their primary tumors and subjected to adjuvant chemotherapy. As the use of specific or pan Akt inhibitors may not be sufficient or may even be detrimental, increasing the levels of Vav1 could be a new approach to improve breast cancer outcomes. This might be particularly relevant for tumors with a triple‐negative phenotype, for which target‐based therapies are not currently available.

**Abbreviations**
DMEM: Dulbecco's modified Eagle's medium
DRFS: distant relapse‐free survival
FBS: fetal bovine serum
GEF: guanosine exchange factor
TMAs: tissue microarrays
TNBC: triple‐negative breast cancers

## 1 Introduction

The multidomain protein Vav1 is physiologically expressed solely in the hematopoietic system in which it participates to cytoskeleton reorganization and gene transcription (Romero and Fischer, 1996; Tybulewicz, 2005). In recent years, aberrant expression of Vav1 has been reported in nonhematopoietic cancers (Bartolome *et al*., 2006; Fernandez‐Zapico *et al*., 2005; Hornstein *et al*., 2003; Lazer *et al*., 2009; Qi *et al*., 2015; Wakahashi *et al*., 2013; Zhu *et al*., 2017), in which this protein is involved in signal transduction processes correlated with tumor phenotype (Fernandez‐Zapico *et al*., 2005; Qi *et al*., 2015; Zhu *et al*., 2017). Vav1 is expressed in the majority of breast carcinomas (Sebban *et al*., 2013), in which we have previously demonstrated its peculiar localization inside the cell nucleus (Grassilli *et al*., 2014). In early‐stage invasive breast tumors, high levels of nuclear Vav1 were positively correlated with low risk of relapse, constituting a positive prognostic factor independent of molecular subtypes. Accordingly, the upmodulation of Vav1 in invasive breast tumor‐derived cells reduced their invasiveness *in vitro* and their metastatic efficiency *in vivo* (Grassilli *et al*., 2014).

The malignancy‐associated role of Vav1 has been variously linked to its best known cytoplasmic function as guanosine exchange factor (GEF) for Rho/Rac GTPases (Bustelo, 2002), mainly devoted to the rearrangement of actin cytoskeleton at the basis of the functional changes that characterize tumor cells (Bartolome *et al*., 2006; Fernandez‐Zapico *et al*., 2005; Lazer *et al*., 2009). However, additional roles of Vav1, correlated to its nuclear localization, were clearly established in cells from myeloid leukemia (Bertagnolo *et al*., 2012; Houlard *et al*., 2002), in which it plays an antitumor role by collaborating in gene expression (Brugnoli *et al*., 2010; Grassilli *et al*., 2016) and in mRNA production and stability (Bertagnolo *et al*., 2011).

In breast tumor‐derived cell lines, divergent effects exerted by Vav1 on Rac1 activation (Sebban *et al*., 2013) suggest the existence of signaling pathways involving Vav1 alternative to guanosine exchange activity. According to its role in modulating nucleic acid metabolism, in mammary tumor cells Vav1 accumulates in subnuclear structures and its downmodulation induces a phenotype‐related expression of genes variously involved in malignant progression (Grassilli *et al*., 2014). They include *Akt1* and the gene encoding for the PI3K/Akt activator PDGFRB (Zhang *et al*., 2007), suggesting that Vav1 may affect the Akt‐related machinery in mammary tumors.

Abnormal activation of Akt pathways is the most common aberrations of signal transduction in solid tumors, including breast cancer (Mundi *et al*., 2016; Yang *et al*., 2016) in which the three known Akt isoforms (Akt1, Akt2, and Akt3) show a phenotype‐related expression (Clark and Toker, 2014; Iacovides *et al*., 2013). Epidemiological and preclinical studies confirmed that activation of Akt is implicated in the pathogenesis of breast cancer also by conferring resistance to systemic treatments (Yang *et al*., 2016) and a number of molecules have been generated to selectively or nonselectively inhibit the three isoforms (Dey *et al*., 2017; Mundi *et al*., 2016). Therefore, despite a promising chance, emerging data revealing different or even opposite functional roles of Akt isoforms in the regulation of proliferation, migration, and invasion (Dillon *et al*., 2009; Li *et al*., 2016; Riggio *et al*., 2017) opened the issue of the reliability of the use of pan or isoform‐specific Akt inhibitors in the different tumor subtypes and of the combined use of inhibitors and cytotoxic chemotherapy. The development of Akt inhibitors is particularly problematic in triple‐negative breast cancers (TNBC) that express all three Akt isoforms and show the highest activation of the Akt downstream pathways (Chin *et al*., 2014; Grottke *et al*., 2016; Massihnia *et al*., 2016).

This work was aimed to establish whether Vav1 has a role in modulating the Akt signaling in breast cancer cell lines belonging to different tumor phenotypes. The study included both *in vitro* and *in vivo* models in which the effects of forcedly modulated Vav1 on the main Akt1‐related pathways were investigated primarily in breast tumor cells with a triple‐negative phenotype. Archived formalin‐fixed breast tumor samples allowed to establish the prognostic significance of the Vav1/p‐Akt relationship in patients with early breast cancer.

## 2 Materials and methods

### 2.1 Antibodies and reagents

All reagents were from Sigma (St Louis, MO, USA) unless otherwise indicated.

For immunochemical analysis, antibodies against Vav1 (sc‐132), Akt1 (sc‐1618), Akt2 (sc‐5270), Akt3 (sc‐11520), p‐Akt1/2/3 (sc‐14032), Bcl‐2 (sc‐509), Caspase‐3 (sc‐371), and IkBα (sc‐7148) were from Santa Cruz Biotechnology (Santa Cruz, CA, USA). Anti‐p‐Akt1 (Ser473) (#4060), anti‐p‐Akt2 (Ser474) (#8599), anti‐p‐P70S6K (Thr389) (#9205), and anti‐P70S6K (#9202) were from Cell Signaling Technology (Danvers, MA, USA). Anti‐Bax (#610983) was from BD Biosciences (Milan, Italy), anti‐Cyclin D1 (#04‐1151) was from Merck Millipore (Milan, Italy), and anti‐β‐Tubulin (#T4026) was from Sigma.

For immunohistochemical analysis, the anti‐Vav1 (sc‐132) and the anti‐Akt1 (sc‐377457) antibodies were from Santa Cruz Biotechnology, anti‐p‐Akt (Ser473) (#3787) antibody was from Cell Signaling Technology, and the anti‐Cyclin D1 (MCP511) antibody was from YLEM (Rome, Italy).

### 2.2 Cell culture

MDA‐MB‐231, MCF7, and MDA‐MB‐453 cell lines were from the American Type Culture Collection (Rockville, MD, USA) and were maintained in Dulbecco's modified Eagle's medium (DMEM, Gibco Laboratories, Grand Island, NY, USA) supplemented with 10% fetal bovine serum (FBS, Gibco Laboratories). The BT‐474 cell line was from ICLC (Genova, Italy) and was cultured in RPMI 1640 growth medium (Gibco Laboratories) supplemented with 10% FBS, 1 mm Na pyruvate, and 0.01 mg·mL^−1^ bovine insulin. Cells were grown at 37 °C in a humidified atmosphere of 5% CO_2_ in air.

MDA‐MB‐231 cells stably expressing Vav1 were obtained by transfection with a construct expressing the human full‐length Vav1, as previously reported (Grassilli *et al*., 2014).

All cell lines were monthly tested for mycoplasm and other contaminations and quarterly subjected to cell identification by single nucleotide polymorphism.

### 2.3 Modulation of Vav1 expression and cell analysis

Vav1 overexpression was obtained by transient transfection with a pEF plasmid expressing the human full‐length Vav1 (Grassilli *et al*., 2014), and the downmodulation of Vav1 was performed by silencing the protein with specific siRNA (Santa Cruz Biotechnology), following previously described procedures (Grassilli *et al*., 2014).

For western blot analysis, total lysates (50 μg protein) and Akt3 immunoprecipitates, obtained as previously reported (Bertagnolo *et al*., 2007), were separated on polyacrylamide denaturing gels, blotted to nitrocellulose membranes (GE Healthcare Life Science, Little Chalfont, UK), and reacted with primary antibodies. Membranes were then incubated with peroxidase‐conjugated secondary antibodies and revealed with the ECL system (PerkinElmer, Boston, MA, USA). The chemiluminescence of bands was acquired with ImageQuant™ LAS 4000 biomolecular imager (GE Healthcare), and the densitometric analysis was performed with image quant tl software (GE Healthcare).

Proliferation of MDA‐MB‐231 cells subjected to forced modulation of Vav1 was evaluated with the xCELLigence Real‐Time Cell Analyzer System (Acea Biosciences Inc., San Diego, CA, USA), as previously reported (Brugnoli *et al*., 2013).

To determine the levels of apoptosis, one‐step staining procedure with the Annexin V‐FITC kit was performed, following manufacturer's protocol (Immunotech, Beckman Coulter Company, Marseille, France). Data collected from 20 000 cells were analyzed using a FACSCalibur flow cytometer (BD Biosciences) with cellquest pro 6.0 analysis software (BD Biosciences).

### 2.4 MDA‐MB‐231‐derived xenografts

All procedures for animal experiments were approved by the Committee on the Use and Care of Animals and carried out in strict accordance with the institution guidelines (CEISA—Interuniversity Ethics Committee for Animal Experimentation, Chieti, Italy). 6 × 10^6^ MDA‐MB‐231 cells, stably overexpressing human Vav1 or an empty vector, were injected subcutaneously into BALB/c nude mice (female, age six weeks, *n* = 10 per group, Charles River Laboratories Italia, Lecco, Italy), anesthetized with ethyl ether. Tumors were weakly measured with caliper, and tumor volume was determined (width^2^ × length)/2).

Throughout the experiments, mice were maintained with free access to pellet food and water. After 8 weeks, the mice were euthanized with inhalation of CO_2_ and the tumor xenografts were removed, photographed, and fixed with 4% paraformaldehyde**.**

Paraffin‐embedded xenografts were sliced into 5‐μm sections using a Leica microtome (Leica Biosystems, Wetzlar, Germany). Tissue sections were deparaffinized, stained with hematoxylin and eosin (HE) dyes (Bio‐Optica, Milano, Italy), and observed by an optical microscope (Carl Zeiss Axiophot 100, Zeiss, Göttingen, Germany) equipped with a Nikon Digital Sight DS Vi1 camera (Nikon Instruments S.p.A., Florence, Italy).

### 2.5 Patients and tumors

The current study included a cohort of 126 primary unilateral infiltrating breast cancers from N0 patients with T1/T2 tumors and diagnosed between 1994 and 2001 at the Regina Elena National Cancer Institute (Rome, Italy). The study was reviewed and approved by the ethics committee of the ‘Regina Elena’ National Cancer Institute, and written informed consent was obtained from all patients, according to the Helsinki Declaration of 1975. Tumors were evaluated for their proliferation levels and receptors status as previously reported (Lattanzio *et al*., 2013). Radiotherapy was offered to all patients, 37 of them were treated exclusively with hormonal therapy and 76 received adjuvant chemotherapy followed or not by hormonal therapy. Patients with HER‐2‐positive tumors did not receive trastuzumab, because it was unavailable during the study period. The median follow‐up was of 94 months (range 19–171 months). Follow‐up data were obtained from institutional records or by the referring physicians. Patients and tumor characteristics are summarized in Table 1. Tissue microarrays (TMAs) were constructed as described by Lattanzio *et al*. (2013).

**Table 1:** Patients and tumor characteristics (*n* = 126)

Variable	Value (%)
Age at diagnosis (years)	
Median	55.7
<50	42 (33.3)
50–65	50 (39.7)
>65	34 (27.0)
Menopausal status	
Pre/perimenopausal	44 (34.9)
Postmenopausal	82 (65.1)
Tumor size	
2 cm	81 (64.3)
>2 cm	45 (35.7)
Histotypes	
Ductal carcinoma	100 (79.4)
Lobular carcinoma	17 (13.5)
Other	9 (7.1)
Molecular subtypes	
Luminal A‐like	56 (44.4)
Luminal B‐like (HER2 negative)	31 (24.6)
Luminal B‐like (HER2 positive)	11 (8.7)
HER2 positive (non‐luminal)	6 (4.8)
Triple negative (ductal)	22 (17.5)
Tumor grade	
1	16 (12.7)
2	68 (54.0)
3	42 (33.3)
Ki‐67	
Low	68 (54.0)
High	58 (46.0)
Patient outcome	
Without recurrence	103 (81.7)
Local recurrence	8 (6.4)
Distant recurrence	15 (11.9)

### 2.6 Immunohistochemical analysis and statistical methods

Immunohistochemical analysis of Vav1 in TMAs and of Akt1 and p‐Akt (Ser473) in xenografts was performed as previously reported (Grassilli *et al*., 2014). For evaluation of Vav1 levels, the cellular staining intensity was estimated with the imagescope software (Aperio, Vista, CA, USA) by analysis of acquired images, as previously described (Grassilli *et al*., 2014). For quantification of Akt1 and p‐Akt (Ser473) staining, optical microscope images containing at least 100 cells were analyzed using the Aperio Positive Pixel Count algorithm, embedded in the aperio imagescope software.

Immunohistochemical analysis of p‐Akt (Ser473) and Cyclin D1 in TMAs was performed as reported by Lattanzio *et al*. (2013). p‐Akt (Ser473) score was based on the number of stained cells: 0, no staining; 1+, < 20% of positive cells; 2+, 20–50% of positive cells; 3+, >50% of positive cells. For statistical purposes, scores of 2+ or 3+ in either the nucleus or cytoplasm were considered high staining (Huang *et al*., 2013). The cutoff value used for high Cyclin D1 expression was >10% (Ravikumar and Ananthamurthy, 2014).

Comparisons of immunostaining (immunohistochemistry, western blot, flow cytometry) values, proliferation rate, and tumor growth between groups were performed using the two‐tailed *t*‐test for unpaired data. The statistical analysis was performed with the graphpad prism 6.0 statistical package (GraphPad Software, San Diego, CA, USA). *P* < 0.05 was considered statistically significant.

The relationship between Vav1 status and the expression of p‐Akt (Ser473) and/or Cyclin D1 in patients’ samples, as well as the correlation of Vav1 with clinicopathological parameters, was evaluated by Pearson's chi‐square test. Kaplan–Meier plots were used to show the survival in specified cohorts and the log‐rank test to check for equality of survival curves. Distant relapse‐free survival (DRFS) was the time from surgery to the occurrence of relapse at a distant site. spss version 15.0 (SPSS, Chicago, IL, USA) was used throughout.

## 3 Results

### 3.1 Modulation of Vav1 affects the activation status of Akt1 in breast cancer‐derived cells with a triple‐negative phenotype

**Figure 1:**
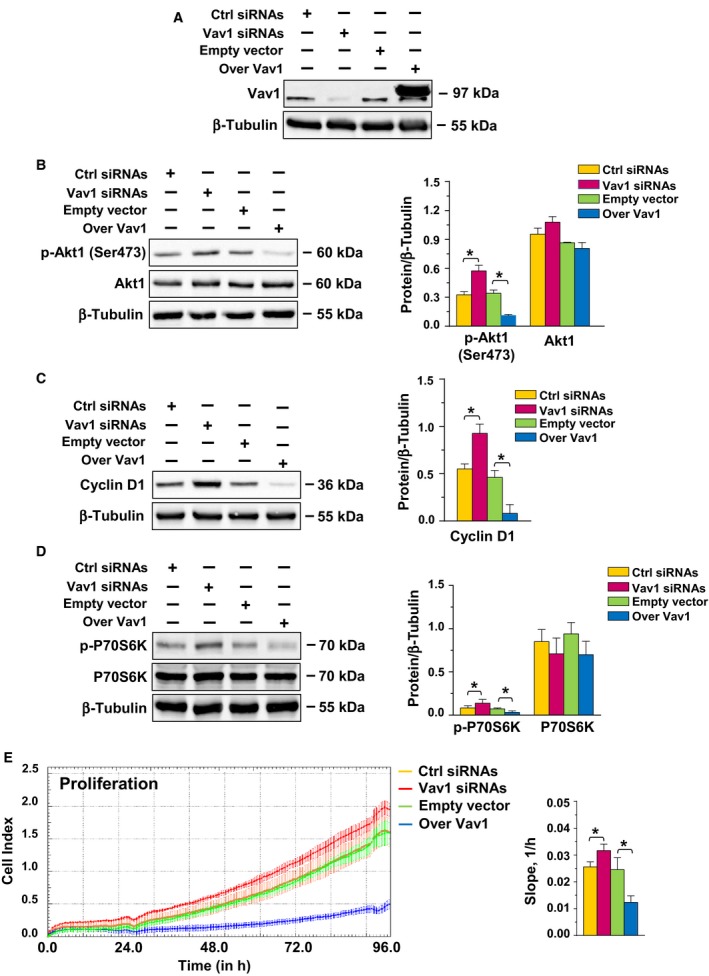
Vav1‐dependent regulation of Akt1 in triple‐negative breast cancer‐derived cells. (A, B, C, D) Representative western blot analysis, performed with the indicated antibodies, of total lysates from MDA‐MB‐231 cells transfected with siRNA specific for Vav1 (Vav1 siRNA) or with a plasmid expressing human Vav1 (Over Vav1). Scramble siRNA (Ctrl siRNA) and an empty vector were used as control of the experiment. β‐Tubulin was blotted as an internal control of loaded proteins. Right histograms show the levels of the indicated proteins normalized to β‐tubulin. (E) MDA‐MB‐231 cells under the same experimental conditions were subjected to dynamic monitoring of proliferation using the impedance‐based xCELLigence Real‐Time Cell analysis (RTCA) system. Cell Index (CI) is reported, and error bars indicate ±SD. The correspondent slope analysis that describes the steepness, incline, gradient, and changing rate of the CI curves over time is shown on the right. All the data are the mean of three separate experiments ±SD. *P < 0.05.

To assess whether Vav1 affects expression and/or activation of Akt1 in breast tumor cells with a triple‐negative phenotype, MDA‐MB‐231 cells were transiently transfected with siRNA specific for Vav1 or with a construct expressing the human protein (Fig. 1A). While immunochemical analysis showed unchanged Akt1 amount, the use of a specific phospho‐antibody revealed, in MDA‐MB‐231 cells subjected to Vav1 downmodulation, an increased phosphorylation of Akt1 on Ser473, known to be required for its maximal activity (Guo *et al*., 2014). Accordingly, a significant decrease in p‐Akt1 (Ser473) was detected as a consequence of Vav1 overexpression (Fig. 1B).

As activated Akt1 exerts its role in tumor progression mainly by inducing cell proliferation or preventing apoptosis (Guerrero‐Zotano *et al*., 2016), the effects of the forced modulation of Vav1 on these cellular events were investigated. Immunochemical analysis revealed a significant increase or decrease in Cyclin D1 as a consequence of silencing or overexpression of Vav1, respectively (Fig. 1C). Lysates from MDA‐MB‐231 cells in which the expression of Vav1 was forcedly modulated were also subjected to immunochemical analysis of the serine/threonine kinase P70S6K. While the total amount of the protein was not affected, its phosphorylation level on Thr389 was significantly increased or decreased, respectively, by silencing or overexpression of Vav1 (Fig. 1D). The real‐time proliferation assay demonstrated a small but significant increase in growth of Vav1‐silenced cells and a substantial decrease in cell proliferation as a consequence of Vav1 overexpression (Fig. 1E).

Immunochemical analysis did not show changes of Bcl‐2, Bax, pro‐Caspase 3, and IkBα (Fig. S1A,B) as a consequence of Vav1 modulation. Accordingly, no significant modification of the number of apoptotic cells was observed (Fig. S1C).

**Figure 2:**
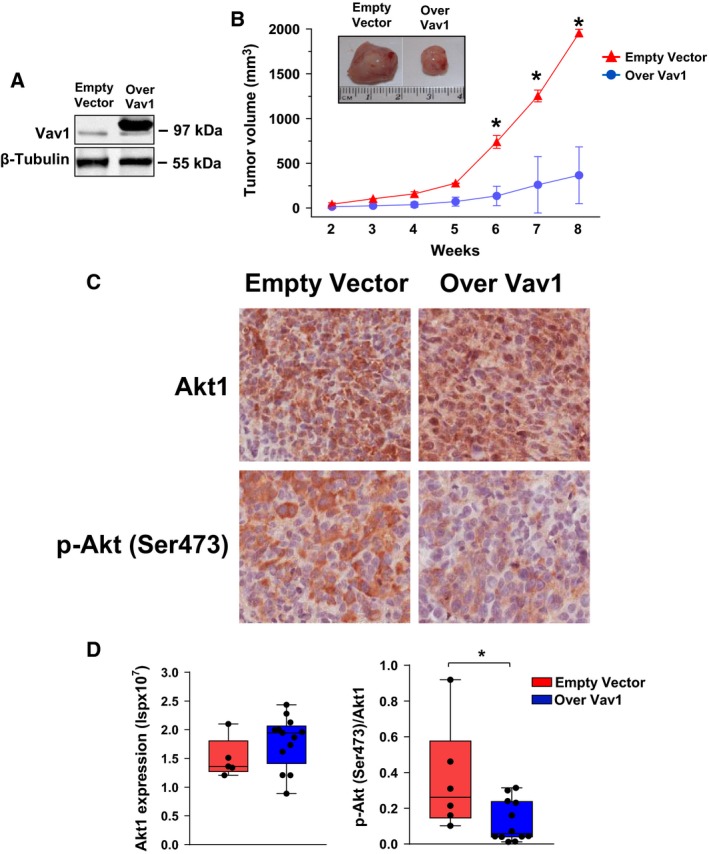
Effects of Vav1 on growth of MDA‐MB‐231‐derived xenografts. (A) Immunochemical analysis with specific antibodies of MDA‐MB‐231 cells stably expressing an empty vector or human Vav1 (Over Vav1) that were injected into 6‐week‐old female nude mice. (B) Xenograft volumes measured from the second week after the injection of MDA‐MB‐231 cells whose Vav1 expression is shown in (A). In (C), representative images of xenograft sections subjected to immunohistochemical analysis with the indicated antibodies (Bar = 50 μm). Positive pixel analysis of Akt1 and p‐Akt (Ser473) staining was carried out using Aperio Positive Pixel Count algorithm and is reported as intensity of strong positive pixels (Isp) (D). *P <* *0.05.

As *in vitro* data suggested that dysregulation of the Akt1 pathway induced by Vav1 is mainly reflected by cell proliferation, the role of Vav1 in affecting the *in vivo* proliferation of MDA‐MB‐231 cells was investigated. Xenografted mice were obtained with MDA‐MB‐231 cells stably overexpressing Vav1 (Fig. 2A), and the solid tumors formed in the subcutaneous skin layer were evaluated. Tumor masses, detected starting from the second week after injection, reached a significantly lower dimension in mice receiving MDA‐MB‐231 cells overexpressing Vav1 compared to those derived from MDA‐MB‐231 transfected with an empty vector (Fig. 2B).

Immunohistochemical analysis of xenografts revealed a similar expression of total Akt1 and a significantly lower level of p‐Akt (Ser473) in tumors derived from mice injected with MDA‐MB‐231 stably overexpressing Vav1 (Fig. 2C,D).

### 3.2 Modulation of Vav1 induces phenotype‐related changes of Akt1 in breast cancer‐derived cells

**Figure 3:**
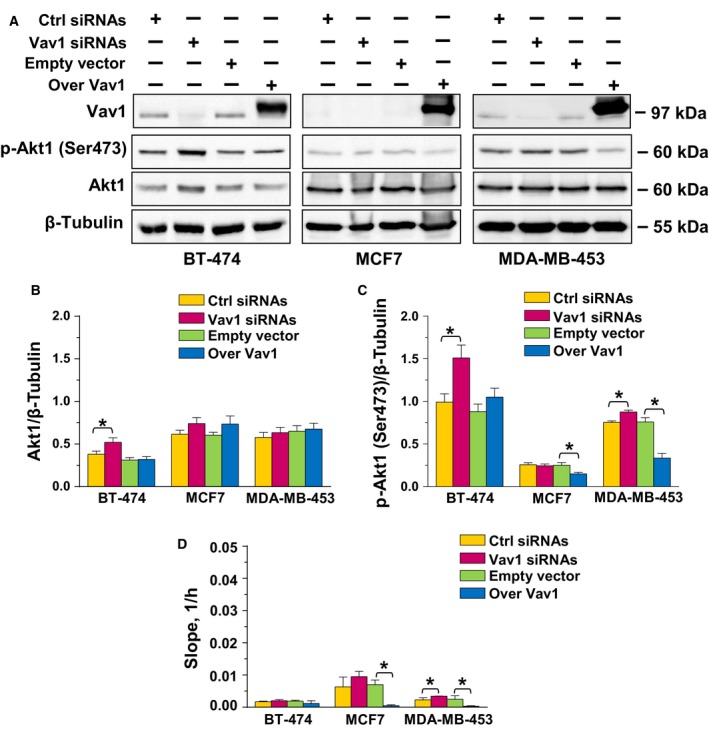
Vav1‐dependent regulation of Akt1 in breast cancer‐derived cells with different phenotypes. (A) Representative western blot analysis, performed with the indicated antibodies, of lysates from BT‐474, MCF7, and MDA‐MB‐453 cells transfected for 48 h with siRNA specific for Vav1 (Vav1 siRNA) or with a construct expressing human Vav1 (Over Vav1). Scramble siRNA (Ctrl siRNA) and an empty vector were used as controls of the experiment. β‐Tubulin was used as internal control for equivalence of loaded proteins. (B, C) Histograms, as deduced from the densitometry of western blot bands, reporting the levels of proteins normalized to β‐tubulin. (D) Slope analysis of the dynamic monitoring of proliferation of BT‐474, MCF7, and MDA‐MB‐453 cells, under the above reported experimental conditions. All the data are the mean of three separate experiments ±SD. *P <* *0.05.

The question of whether Vav1 affects Akt1 activation in breast tumor cells with phenotypes other than triple negative was addressed in the breast cancer‐derived cell lines BT‐474, MCF7, and MDA‐MB‐453, which represent the most frequent subtypes of breast tumors and express Vav1 to a variable extent (Grassilli *et al*., 2014; Fig. 3A). As reported in Fig. 3, the silencing of Vav1 induced increase in Akt1 expression only in BT‐474 cells (Fig. 3A, 3B), consistent with our previous data in the same cell model (Grassilli *et al*., 2014) and in parallel with the increased p‐Akt1 (Ser473) levels (Fig. 3A,C). Silencing of Vav1 also induced increase in p‐Akt1 (Ser473) in MDA‐MB‐453 cells, while no effects were observed in MCF7, according to the very low basal expression of the protein in this cell line (Fig. 3A,C). The total protein amount remained unchanged (Fig. 3A,B), while a significant decrease in the p‐Akt1 (Ser473) levels was induced by Vav1 overexpression in both MCF7 and MDA‐MB‐453 cell lines (Fig. 3A,C).

The analysis of cell proliferation failed to reveal changes in growth of BT‐474 subjected to forced modulation of Vav1 but indicated that silencing of this protein is sufficient to increase the growth of MDA‐MB‐453 and that Vav1 overexpression induced a decline of proliferation in both MCF7 and MDA‐MB‐453 cells (Fig. 3D), paralleling the effects observed on p‐Akt1 (Ser473) levels.

### 3.3 Silencing of Vav1 induced the expression but not the activation of Akt2 in breast tumors cells with different phenotypes

**Figure 4:**
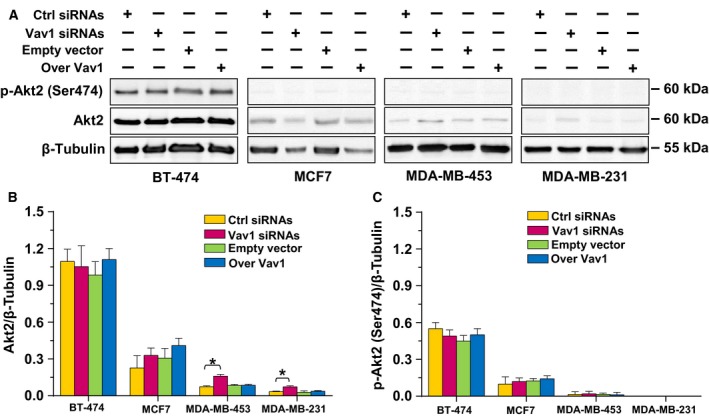
Vav1‐dependent regulation of Akt2 in breast cancer‐derived cells with different phenotypes. (A) Representative western blot analysis, performed with the indicated antibodies, of lysates from BT‐474, MCF7, MDA‐MB‐453, and MDA‐MB‐231 cells transfected for 48 h with siRNA specific for Vav1 (Vav1 siRNA) or with a construct expressing human Vav1 (Over Vav1). Scramble siRNA (Ctrl siRNA) and an empty vector were used as controls of the experiment. β‐Tubulin was used as internal control for equivalence of loaded proteins. (B, C) Histograms, as deduced from the densitometry of western blot bands, reporting the levels of proteins normalized to β‐tubulin. All the data are the mean of three separate experiments ±SD. *P <* *0.05.

Concerning the possible involvement of Vav1 in expression and/or activation of Akt isoforms other than Akt1, the use of specific antibodies revealed a significant increase in Akt2 expression in the MDA‐MB‐453 and MDA‐MB‐231 cells in which Vav1 was silenced, while the protein level remained unchanged in BT‐474 and MCF7 cells (Fig. 4A,B). No significant levels of Akt2 phosphorylated on Ser474 were revealed in all cell lines grown in control conditions or subjected to forced modulation of Vav1 (Fig. 4A,C).

**Figure 5:**
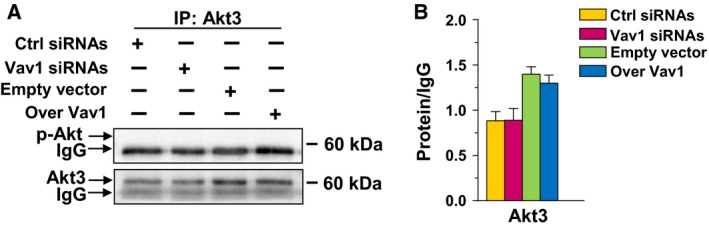
Vav1 and Akt3 in MDA‐MB‐231 cells. (A) Representative western blot analysis of Akt3 immunoprecipitated from MDA‐MB‐231 cells transfected for 48 h with siRNA specific for Vav1 (Vav1 siRNA) or with a construct expressing human Vav1 (Over Vav1) and revealed with an anti‐p‐Akt or with a specific anti‐Akt3 antibody. IgG was blotted as a control for equal antibody addition to samples. The normalized values of Akt3 are shown in the right (B). All the data are the mean of three separate experiments performed in triplicate ±SD.

Concerning Akt3, literature data excluded its expression (Mundi *et al*., 2016) and our results failed to reveal effects of Vav1 modulation on this Akt isoform in BT‐474, MCF7, and MDA‐MB‐453 cell lines (data not shown). In MDA‐MB‐231 cells, known to express Akt3, no difference in protein expression and no phosphorylation were revealed as a consequence of forced modulation of Vav1 (Fig. 5).

### 3.4 The relationship between Vav1 and phosphorylation status of Akt has a prognostic significance in invasive breast tumors

Once established that Vav1 modulates the activation of Akt1 in breast tumor‐derived cells, we investigated the association between the Vav1/p‐Akt (Ser473) levels and the clinical outcome of breast cancer patients. The study group consisted of 126 patients with primary unilateral breast carcinoma (T1/T2), with no evidence of nodal involvement and distant metastases (Table 1), in which both Vav1 and phosphorylated Akt were evaluated by immunohistochemistry on TMAs. The selection of the cutoff value for Vav1 was based on the results of a previously reported study (Grassilli *et al*., 2014). Based on this dichotomization, 60 cases of 126 (47.6%) were included in the group of low Vav1‐expressing tumors and 66 (52.4%) were included in the group of high Vav1‐expressing tumors. The levels of activated Akt were dichotomized as reported in Materials and Methods, and the percentage of cases with low or high levels of p‐Akt (Ser473) was 69.8% and 30.2%, respectively.

By Pearson's chi‐square analysis, Vav1 expression was found to be inversely correlated with p‐Akt staining (*P = *0.011*; R = *–0.239), with 80.3% of Vav1^high^ tumors that displayed low levels of phosphorylated Akt (Table 2). A strong inverse correlation (*P < *0.001*; R = *–0.544) was also found between Cyclin D1 expression and Vav1/p‐Akt (Ser473) status. In particular, 74.5% of tumors with Vav1^high^/p‐Akt^low^ expressed low levels of Cyclin D1, while 83.3% of Vav1^low^/p‐Akt^high^ cases showed high Cyclin D1 staining (Table 3).

**Table 2:** Correlation between Vav1 expression and p‐Akt (Ser473) status in breast cancer patients (*n* = 126)

Variable	Vav1^low^, *n* (%)	Vav1^high^, *n* (%)	*P* a	*R* b
p‐Akt^low^	35 (58.3)	53 (80.3)	0.011	−0.239
p‐Akt^high^	25 (41.7)	13 (19.7)		

a Pearson's chi‐square test.

b Pearson's *R*.

**Table 3:** Correlation between Vav1/p‐Akt (Ser473) and Cyclin D1 expressions in breast cancer patients (*n* = 75)

Variable	Vav1^low^/p‐Akt^high^ *n* (%)	Vav1^high^/p‐Akt^low^ *n* (%)	*P* a	*R* b
Cyclin D1^Low^	4 (16.7)	38 (74.5)	<0.001	−0.544
Cyclin D1^High^	20 (83.3)	13 (25.5)		

a Pearson's chi‐square test.

b Pearson's R; °Cutoff value for Cyclin D1 > 10%.

**Figure 6:**
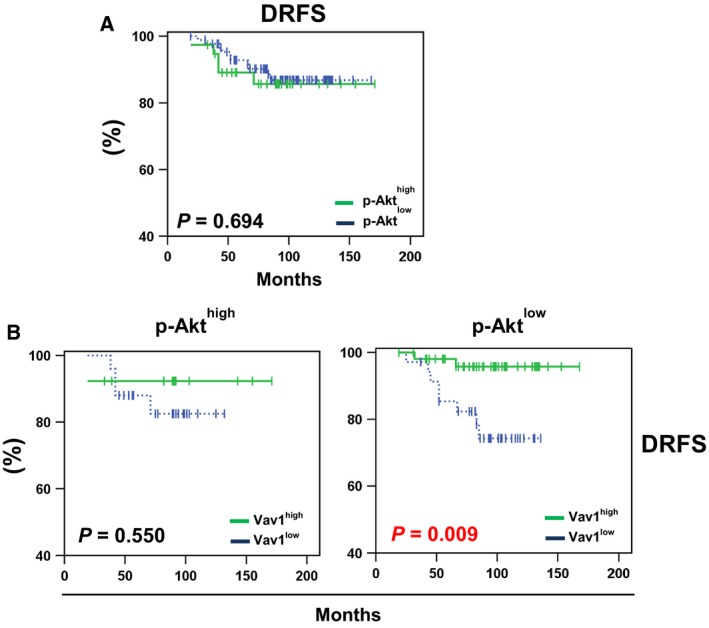
Distant relapse‐free survival of breast cancer patients according to p‐Akt and/or Vav1 status of their primary tumors. (A) Kaplan–Meier estimates of distant relapse‐free survival (DRFS) in patients (*n* = 126) according to p‐Akt status. (B) Kaplan–Meier estimates of DRFS in patients with p‐Akt^high^ tumors (*n* = 38) (left panel), and in patients with p‐Akt^low^ tumors (*n* = 88) (right panel), according to high (green solid line) and low (blue dashed line) expression of Vav1. In these cohort of patients, a distant relapse occurred in the 11% (10/88) p‐Akt^low^ and 13% (5/38) p‐Akt^high^ tumors, in the 16% (4/25) Vav1^low^ and 8% (1/13) Vav1^high^ of patients with p‐Akt^high^ tumors, and in the 23% (8/35) Vav1^low^ and 4% (2/53) Vav1^high^ of patients with p‐Akt^low^ tumors.

At Kaplan–Meier analysis, the p‐Akt (Ser473) status was not associated with distant relapse in patients of the whole population (Fig. 6A). Only in patients harboring p‐Akt^low^ tumors, we found that low levels of Vav1 were significantly associated with higher incidence of distant relapse (*P = *0.009) (Fig. 6B).

To explore the possible predictive value of Vav1 in patients with p‐Akt^low^ tumors, analyses of DRFS were conducted on the subset of 88 patients receiving systemic adjuvant treatment (chemotherapy or hormonal). At univariate analyses, low expressions of Vav1 were significantly associated with higher risk of metastases (HR = 8.2; 95% CI = 1.0–66.8; *P* = 0.049) only in patients treated with chemotherapy (Table 4). In the cohort of patients with p‐Akt^low^ tumors, we also searched for possible correlations among the expression of Vav1 and the analyzed clinic‐pathological variables. As reported in Table 5, Vav1 was significantly less expressed in tumors from patients with age <50 at diagnosis (*P = *0.018), while Vav1 status did not differ significantly for the distribution of the other considered clinic‐pathological variables.

**Table 4:** Risk of distant relapse associated with Vav1 status in p‐Akt (Ser473)^low^ breast cancer cases (*n* = 88) according to adjuvant therapy

Adjuvant therapy	HR^a^	95% CI	*P* b
Radiotherapy (*n* = 10)	1.4	0.1–1.0	0.582
Hormonal (*n* = 31)	1.0	0.1–2.0	0.608
Chemotherapy (*n* = 47)	8.2	1.0–66.8	0.049

a Hazard ratio of low versus high Vav1 expression.

b Pearson's chi‐square test.

**Table 5:** Vav1 status according to clinicopathological features of breast cancer patients with p‐Akt^low^ tumors (*n* = 88)

Variable	Vav1		*P* a
	Low, *n* (%)	High, *n* (%)	
Age at diagnosis (years)			
<50	15 (42.9)	10 (18.9)	0.018
50–65	9 (25.7)	28 (52.8)	
>65	11 (31.4)	15 (28.3)	
Menopausal status			
Pre/perimenopausal	15 (42.9)	12 (22.6)	0.054
Postmenopausal	20 (57.1)	41 (77.4)	
Tumor size			
≤2 cm	25 (71.4)	35 (66.0)	0.646
>2 cm	10 (28.6)	18 (34.0)	
Histotypes			
Ductal carcinoma	27 (77.1)	46 (86.8)	0.466
Lobular carcinoma	5 (14.3)	5 (9.4)	
Other	3 (8.6)	2 (3.8)	
Molecular subtypes			
Luminal A‐like	18 (51.4)	23 (43.4)	0.260
Luminal B‐like (HER2 negative)	6 (17.1)	15 (28.3)	
Luminal B‐like (HER2 positive)	1 (2.9)	4 (7.5)	
HER2 positive (nonluminal)	2 (5.7)	0 (0.0)	
Triple negative (ductal)	8 (22.9)	11(20.8)	
Tumor grade			
1	6 (17.1)	6 (11.3)	0.478
2	16 (45.8)	31 (58.5)	
3	13 (37.1)	16 (30.2)	
Ki‐67			
Low	22 (62.9)	31 (58.5)	0.824
High	13 (37.1)	22 (41.5)	

a Pearson's chi‐square test.

## 4 Discussion

Currently, breast cancer treatments are highly dependent on tumor phenotypes and mainly developed to target ER, PR, or HER2. As a result, systemic adjuvant chemotherapy is the only option for TNBC patients who, although highly responsive, show a greater recurrence and poorer prognosis than those bearing other types of breast cancers (Fleisher *et al*., 2016). The PI3K/Akt/mTOR pathway is an exciting objective for developing anticancer strategies, and a number of agents targeting different members of this signaling pathway have been developed (Chia *et al*., 2015; Ma, 2015; Nagini, 2017). Unfortunately, frequent mutations or copy number changes in genes important for cell cycle arrest or apoptosis limit the antitumor activity of inhibitors of the PI3K/Akt pathway (Ma, 2015). The scenario is further complicated by the expression of all three Akt isoforms in cells with a triple‐negative phenotype, with dissimilar effects on the metastatic process (Dillon *et al*., 2009; Grottke *et al*., 2016; Riggio *et al*., 2017), raising the problem of the identification of isoform‐specific inhibitors to be selectively used in the different breast tumor subtypes.

Our previous data revealed that Vav1, ectopically expressed in breast tumors in which positively correlates with follow‐up, regulates the transcription levels of genes encoding for Akt1 in breast tumor‐derived cells with a luminal B phenotype and for activators of the PI3K/Akt signaling in TNBC‐derived cells (Grassilli *et al*., 2014). To elucidate whether modulation of the complex pathways triggered by Akt may be at the basis of the role of Vav1 in breast cancer cells, we explored here the ability of this protein to affect Akt expression and/or activation in breast tumor cells with different phenotypes. We firstly investigated the role of Vav1 in regulating Akt1 in TNBC‐derived MDA‐MB‐231 cells, confirming our previous data indicating that the forced modulation of Vav1 is unable to affect the expression of Akt1 (Grassilli *et al*., 2014). Remarkably, we found that silencing of Vav1 increased the phosphorylation level of Akt1 at Ser473, known to be required for its maximal activity (Guo *et al*., 2014). By investigating *in vitro* the main events triggered by activated Akt1, we demonstrated that the downmodulation of the p‐Akt1 (Ser473) level induced by Vav1 in MDA‐MB‐231 cells correlates with their reduced proliferation rate, possibly mediated by the Akt/S6K pathway, a well‐described mechanism in breast tumor cells (Riggio *et al*., 2017). The reduced size of xenografts derived from MDA‐MB‐231 stably overexpressing Vav1 confirmed the *in vivo* role of this protein as a strong suppressor of Akt1 activity in cells with a TNBC phenotype.

The ability of Vav1 to downmodulate p‐Akt1 is not restricted to cells with a triple‐negative phenotype, as we revealed by modulating Vav1 in cell lines representing the most frequent breast tumor subtypes. Apart from BT‐474 cells, showing a luminal B phenotype, in which we confirmed the increase in Akt1 expression as a consequence of Vav1 downmodulation (Grassilli *et al*., 2014), overexpression of Vav1 reduced p‐Akt1 (Ser473) levels in MCF‐7 cells, showing a luminal A phenotype, and in the HER2‐positive MDA‐MB‐453, allowing to conclude that, in breast tumor‐derived cells and regardless their phenotype, Vav1 is part of signaling pathways ended to modulate the activation status of Akt1.

We further explored the possible role of Vav1 in modulating Akt2, whose expression is phenotype‐dependent and whose role in breast tumors seems to be different from that of Akt1 and mainly associated with migration and metastasis (Chin *et al*., 2014; Dillon *et al*., 2009). Although all used cell lines express Akt2, we found that silencing of Vav1 induced a slight expression of Akt2 only in MDA‐MB‐453 and MDA‐MB‐231 cells, without affecting its phosphorylation at the Ser474 residue and, therefore, its activation status (Vadlakonda *et al*., 2013). As no effects on Akt3 expression and/or phosphorylation were observed by modulating Vav1, a phenotype‐related mechanism in which the removal of Vav1 releases transcriptional machineries that regulate the expression of specific Akt isoforms may be postulated. This may involve the estrogen receptors, already reported to influence the expression of the different Akt isoforms (Sun *et al*., 2001; Yndestad *et al*., 2017).

Overall, our data indicate that, in breast tumor‐derived cells with different phenotypes, the sole modulation of Vav1 is sufficient to affect the activation status of Akt1, involved in the insurgence and growth of breast tumors, but not of Akt2, mainly responsible of tumor metastasis (Dillon *et al*., 2009; Riggio *et al*., 2017). These data also suggest that, only in breast tumor cells with an ER‐negative phenotype, low Vav1 levels promote high expression of Akt2 that may be recruited and activated by specific environmental events.

The relationship between cellular staining of Vav1 and activated Akt was also investigated in the tumor tissue of 126 patients with completely resected (T1‐T2, N0) breast cancer, showing that more than 80% of tumors expressing high levels of Vav1 displayed low levels of phosphorylated Akt. Also in tumor tissues, we found that high levels of Vav1 and low levels of p‐Akt correlated with low Cyclin D1 staining, suggestive of the inverse relationship between Vav1 and activated Akt1. By performing a retrospective analysis, we found that, at variance with the sole p‐Akt status, the Vav1/p‐Akt relationship may have a prognostic significance. Remarkably, Vav1 levels significantly influence the follow‐up of patients with low p‐Akt in their primary tumors, in which low expression of Vav1 is associated with the highest probability of distant relapses. These results may be explained considering that Akt1, the only Akt isoform activated by low Vav1 in cell lines with different phenotypes, may inhibit EMT (Li *et al*., 2016) and acts as an invasion suppressor (Riggio *et al*., 2017). Accordingly, p‐Akt^low^ tumors, lacking these protective mechanisms, show the greatest dependence on Vav1 levels that we demonstrated to inversely correlate with the expression of EMT‐related genes (Grassilli *et al*., 2014). In addition, we revealed that the higher incidence of distant relapses only occurs in patients with p‐Akt^low^/Vav1^low^ tumors treated with adjuvant chemotherapy, indicative of the critical role of the balance between expression of Akt and p‐Akt (Ser473) in breast cancer chemo‐resistance and metastasis (Xu *et al*., 2018). Finally, we found that tumors with p‐Akt^low^/Vav1^low^, showing the poorer prognosis, were correlated with a significantly lower age at diagnosis, allowing to identify in younger patients the main potential beneficiaries of the evaluation of p‐Akt/Vav1 relationship.

## 5 Conclusions

On the basis of our results, we can conclude that, in breast tumor‐derived cells, Vav1 is responsible of downmodulating Akt, acting at expression and/or activation levels of the different isoforms depending on tumor subtype. The p‐Akt/Vav1 relationship may have a prognostic role, and low Vav1 is a negative prognostic parameter in node‐negative breast cancer with low p‐Akt. As in breast cancer, the use of specific or pan Akt inhibitors may not be sufficient or may even be detrimental as it might promote tumor invasiveness and cancer dissemination, protocols devised to increase the levels of Vav1 could be an option to improve breast cancer outcomes. This might be particularly relevant for tumors with a triple‐negative phenotype, for which target‐based therapies are not currently available.

## Authors’ contributions

VB was responsible for the study concept, supervised all the experiments, and integrated the results. SG and FB performed *in vitro* experiments and assisted with the data analysis and interpretation. AB provided technical and material support. MM performed *in vivo* experiments. SG and RL performed tumor staining. RL performed survival and statistical analysis. LP provided patients samples. MP provided patients’ associated clinical data. SC critically revised the manuscript for important intellectual content. VB drafted the manuscript with input and approval from all authors.

## Supporting information


**Fig. S1.** Effects of Vav1 on Akt mediated apoptosis of MDA‐MB‐231 cells.
