# Supplementary material for: Vav1 downmodulates Akt in different breast cancer subtypes: a new promising chance to improve breast cancer outcome
Source: Mol Oncol. 2018 May 16;12(7):1012–25. doi: 10.1002/1878-0261.12203 (PMC6026867; doi:10.1002/1878-0261.12203)
Supplement: Supplementary file 1 — Fig. S1. Effects of Vav1 on Akt mediated apoptosis of MDA‐MB‐231 cells. [file MOL2-12-1012-s001.pdf]

Supplementary Figure 1. Effects of Vav1 on Akt mediated apoptosis of MDA-MB-231 cells.

(A) Representative Western blot analysis with the indicated antibodies of lysates from MDA-MB-231 cells transfected with siRNAs specific for Vav1 (Vav1 siRNAs) or with a construct expressing human Vav1 (Over Vav1). Scramble siRNAs (Ctrl siRNAs) and an empty vector were used as control of the experiment. (B) Histograms, as deduced from the densitometry of Western blot bands, reporting the levels of proteins normalized to ϐ-Tubulin. All the data are representative of 3 separate experiments. (C) MDA-MB-231 cells, cultured under the above reported experimental conditions, were double-stained with a solution containing Annexin V-FITC and Propidium Iodide (PI) and subjected to cytometrical analysis. This assay was performed to differentiate between viable (Annexin-Vī /PIī , lower left), early apoptotic (Annexin-V+/PIī , lower right), late apoptotic (Annexin-V+/PI+, upper right) and necrotic (Annexin-Vī /PI+, upper left) cells. The expression of Annexin V-FITC and PI was presented on a bi-parametric dot plot in which the percentage of cells in early and late apoptosis is indicated.

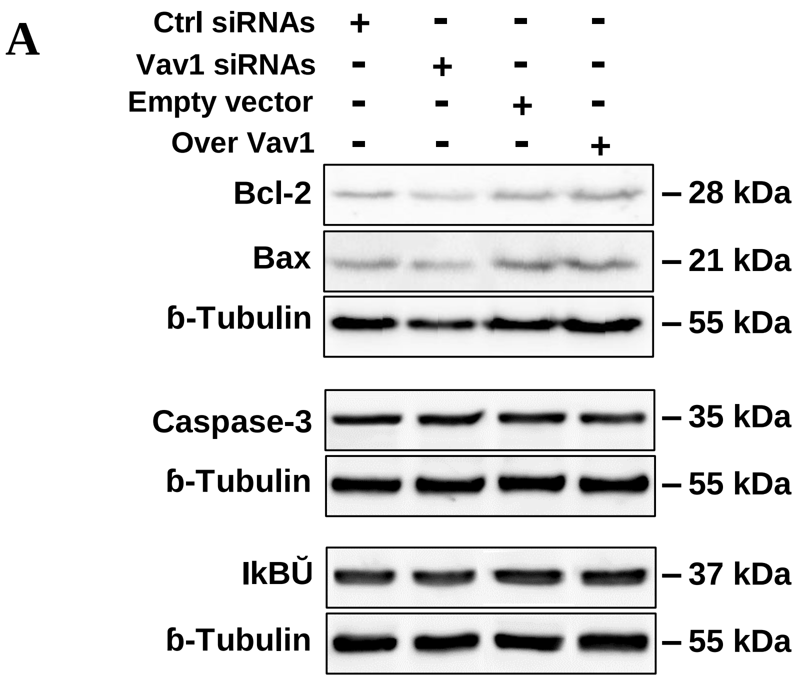

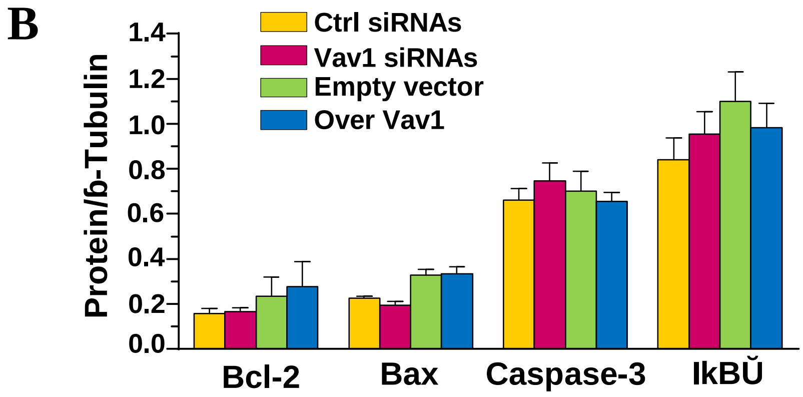
B
Ctrl siRNAs
Vav1 siRNAs
Empty vector
Over Vav1
Protein/ß-Tubulin
1.4
1.2
1.0
0.8
0.6
0.4
0.2
0.0
Bcl-2
Bax
Caspase-3
IkBŬ

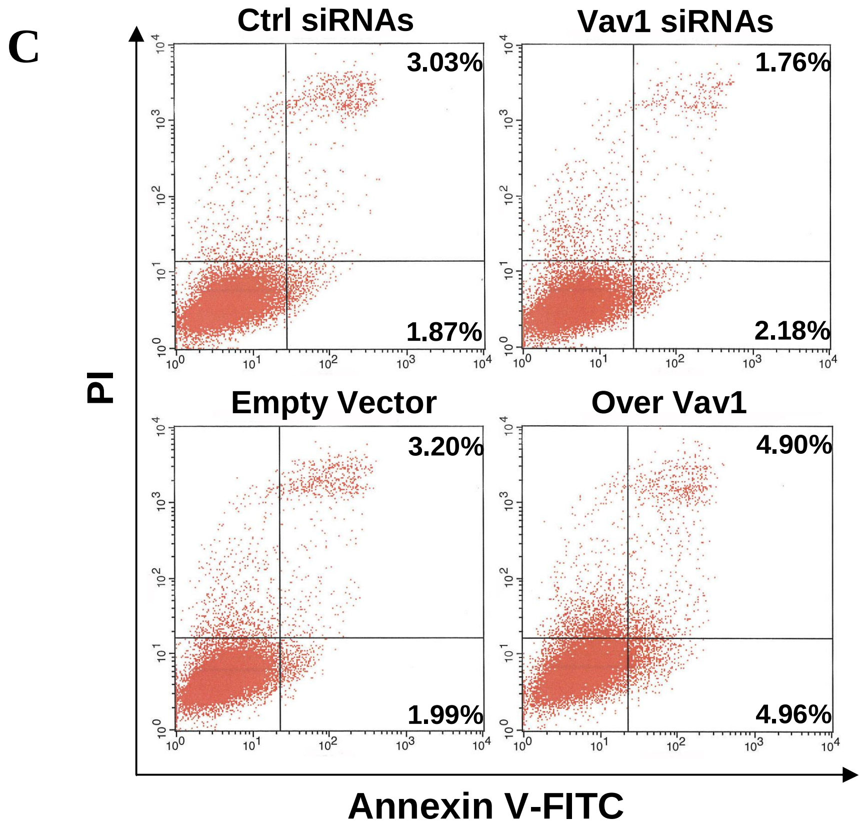
C
Ctrl siRNAs
3.03%
1.87%
Vav1 siRNAs
1.76%
2.18%
Empty Vector
3.20%
1.99%
Over Vav1
4.90%
4.96%
PI
Annexin V-FITC
